# 568 Clinician Survey on Emotional Challenges in Treating Self-Immolation or Self-Inflicted Burn Patients

**DOI:** 10.1093/jbcr/irae036.202

**Published:** 2024-04-17

**Authors:** Ashley E Honea, Karen J Richey, Kevin N Foster

**Affiliations:** Valleywise Health Medical Center - Arizona Burn Center, Phoenix, AZ; Arizona Burn Center Valleywise Health, Phoenix, AZ; Valleywise Health Medical Center - Arizona Burn Center, Phoenix, AZ; Arizona Burn Center Valleywise Health, Phoenix, AZ; Valleywise Health Medical Center - Arizona Burn Center, Phoenix, AZ; Arizona Burn Center Valleywise Health, Phoenix, AZ

## Abstract

**Introduction:**

Self-immolation (SIM) is an uncommon method of attempted suicide involving flammable substances, with suicidal intent. By contrast self-inflicted (SIF) burn injuries utilize a chemical or heated object to cause injury, without suicidal intent. Caring for either patient population can have emotional and ethical challenges. The purpose of this study was to better understand the perspectives and experiences of the healthcare clinician when caring for these patients and identify the need for enhanced support.

**Methods:**

An 11-item survey was distributed to burn center professionals via SurveyMonkey. Questions were developed to seek healthcare professionals’ attitudes, emotional and ethical challenges in treating these patient populations. Respondents were stratified into two groups, those that responded yes to having found it emotionally challenging to care for this patient population (Y) and those that responded no (N).

**Results:**

A total of 65 individuals responded to the survey, and 58 surveys were fully completed. The majority, 65%, of respondents were between ages 22-39, (n=41). Most were burn nurses, 46.7% (n = 29), followed by rehabilitation professionals (rehab), 12.9% (n = 8), advanced practice practitioners (APP), 9.6% (n=6) and physicians 8% (n=5). When stratified, 53% (n=31) responded yes (Y) when asked if they found it emotionally challenging to care for this patient population and 48% (n=28) responded no (N). There were no significant differences between groups for age, gender, years in profession or years in burn care. When examined by profession, 75% of rehab, 67% of psychosocial, 50% of nurses, 40% of physicians and 33% of APP respondents answered yes that care of this population was emotionally challenging. The majority of both groups responded that they received support from team members. When asked if care of these patients created an ethical or moral dilemma 42% of Y and 25% of N responded yes. Y were more likely to have experienced a change in their own mental health 52% vs 7% N. When asked if same day support would be helpful 74% (n=23) of Y and 42% (n=12) responded yes. The preferred format for help was 1:1 or group.

**Conclusions:**

The majority of staff reported emotional and/or ethical challenges when caring for this population created. Of those who responded that care of these patients was not emotionally challenging, interestingly, 25% experienced ethical or moral dilemmas and 43% felt that having same day support would be beneficial. Identification of the need for enhanced support for staff in the hospital setting is critical to avoid burnout, secondary traumatic stress and compassion fatigue for healthcare professionals.

**Applicability of Research to Practice:**

This study highlights the need for a specialized in-house enhanced support program for burn professionals to address their emotional and mental well-being.

